# Supplementary material for: Mechanisms governing the pioneering and redistribution capabilities of the non-classical pioneer PU.1
Source: Nat Commun. 2020 Jan 21;11:402. doi: 10.1038/s41467-019-13960-2 (PMC6972792; doi:10.1038/s41467-019-13960-2)
Supplement: Supplementary file 7 — Source data [file 41467_2019_13960_MOESM7_ESM.zip › Source_Data/Figure6/Figure6B_MotifScanOutput/ETS-specific/homerResults/motif17.similar.html]

motif17

## Information for motif17

C
T
G
A
A
G
C
T
A
G
C
T
A
C
G
T
A
G
T
C
G
A
C
T
G
T
A
C
C
T
G
A
C
T
A
G
G
C
T
A
C
G
T
A
  
Reverse Opposite:  

C
G
A
T
C
G
A
T
A
G
T
C
G
A
C
T
C
A
T
G
C
T
G
A
T
C
A
G
C
G
T
A
C
T
G
A
T
C
G
A
G
A
C
T
  

|  |  |
| --- | --- |
| p-value: | 1e-62 |
| log p-value: | -1.444e+02 |
| Information Content per bp: | 1.634 |
| Number of Target Sequences with motif | 619.0 |
| Percentage of Target Sequences with motif | 8.09% |
| Number of Background Sequences with motif | 1408.6 |
| Percentage of Background Sequences with motif | 3.49% |
| Average Position of motif in Targets | 99.6 +/- 52.3bp |
| Average Position of motif in Background | 98.7 +/- 63.4bp |
| Strand Bias (log2 ratio + to - strand density) | -0.0 |
| Multiplicity (# of sites on avg that occur together) | 1.08 |
| Motif File: | file (matrix) reverse opposite |

### Similar de novo motifs found

|  |  |  |  |  |  |  |  |
| --- | --- | --- | --- | --- | --- | --- | --- |
| Rank | Match Score | Redundant Motif | P-value | log P-value | % of Targets | % of Background | Motif file |
| 1 | 0.979 | A C G T A C G T A C G T A G T C A G C T A T G C C T G A A C T G C G T A C G T A | 1e-57 | -133.279467 | 6.54% | 2.62% | motif file (matrix) |
| 2 | 0.975 | A C G T A C G T A G T C A G C T A C G T C T G A A C T G C G T A C G T A T G C A C G A T T A G C G T C A | 1e-53 | -123.603323 | 6.37% | 2.62% | motif file (matrix) |
| 3 | 0.945 | G A C T G A C T A C G T A G T C G A C T A T C G C T G A T C A G T G C A C T G A T G C A G C A T A T G C G C T A | 1e-52 | -120.306130 | 5.12% | 1.88% | motif file (matrix) |
| 4 | 0.965 | G C A T G C A T G A T C G C A T T A C G C T G A T A C G G C T A C T G A T G C A G A C T A T G C | 1e-50 | -117.189192 | 4.21% | 1.37% | motif file (matrix) |
| 5 | 0.941 | A G C T A C G T A G T C A G C T A T G C C T A G A C T G G C T A C G T A | 1e-47 | -108.504294 | 5.09% | 1.98% | motif file (matrix) |
| 6 | 0.867 | A G C T A C G T T G A C G C A T A T G C C G T A A C T G C G T A | 1e-22 | -52.281077 | 12.83% | 9.05% | motif file (matrix) |
